# Confounding from Cryptic Relatedness in Case-Control Association Studies

**DOI:** 10.1371/journal.pgen.0010032

**Published:** 2005-09-02

**Authors:** Benjamin F Voight, Jonathan K Pritchard

**Affiliations:** Department of Human Genetics, University of Chicago, Chicago, Illinois, United States of America; University of Michigan, United States of America

## Abstract

Case-control association studies are widely used in the search for genetic variants that contribute to human diseases. It has long been known that such studies may suffer from high rates of false positives if there is unrecognized population structure. It is perhaps less widely appreciated that so-called “cryptic relatedness” (i.e., kinship among the cases or controls that is not known to the investigator) might also potentially inflate the false positive rate. Until now there has been little work to assess how serious this problem is likely to be in practice. In this paper, we develop a formal model of cryptic relatedness, and study its impact on association studies. We provide simple expressions that predict the extent of confounding due to cryptic relatedness. Surprisingly, these expressions are functions of directly observable parameters. Our analytical results show that, for well-designed studies in outbred populations, the degree of confounding due to cryptic relatedness will usually be negligible. However, in contrast, studies where there is a sampling bias toward collecting relatives may indeed suffer from excessive rates of false positives. Furthermore, cryptic relatedness may be a serious concern in founder populations that have grown rapidly and recently from a small size. As an example, we analyze the impact of excess relatedness among cases for six phenotypes measured in the Hutterite population.

## Synopsis

There has long been concern in the human genetics community that case-control association studies may be subject to high rates of false positives if there is unrecognized population structure. After being considered rather suspect in the 1990s for this reason, case-control studies are regaining popularity, and will no doubt be used widely in future genome-wide association studies.

Therefore, it is important to fully understand the types of factors that can lead to excess rates of false positives in case-control studies. Virtually all of the previous discussion in the literature of excess false positives (confounding) in case-control studies has focused on the role of population structure. Yet a widely cited 1999 paper by Devlin and Roeder (that introduced the genomic control concept) argued that, in fact, “cryptic relatedness” (referring to the idea that some members of a case-control sample might actually be close relatives, unbeknownst to the investigator) is likely to be a far more important confounder than population structure. Moreover, one of the two main types of statistical approaches for dealing with confounding in case-control studies (i.e., structured association methods) does not correct for cryptic relatedness.

This work provides the first careful model of cryptic relatedness, and outlines exactly when cryptic relatedness is and is not likely to be a problem. The authors provide simple expressions that predict the extent of confounding due to cryptic relatedness. Surprisingly, these expressions are functions of directly observable parameters. The analytical results show that, for well-designed studies in outbred populations, the degree of confounding due to cryptic relatedness will usually be negligible. However, in contrast, studies where there is a sampling bias toward collecting relatives may indeed suffer from excessive rates of false positives.

## Introduction

Case-control association studies are a popular, convenient, and potentially powerful strategy for identifying genes of small effect that contribute to complex traits [1]. However, case-control studies may be susceptible to high rates of false positives if the underlying statistical assumptions are not satisfied. In particular, it has long been a source of concern that population structure might cause confounding in such studies [2,3], and a number of statistical methods have been developed to detect and correct for unrecognized population structure [4–9].

However, in their 1999 paper, Devlin and Roeder argued that another source of confounding, “cryptic relatedness,” might actually be a more serious source of error for case-control studies. Cryptic relatedness refers to the idea that some members of a case-control sample might actually be close relatives, in which case their genotypes are not independent draws from the population frequencies. When that happens, the allele frequency estimates in the case and control samples are unbiased but may have greater variance than expected, and tests of association that ignore the excess relatedness have inflated type-1 error rates. Devlin and Roeder [4] pointed out that if one is doing a genetic association study, then one surely believes that the disease has an underlying genetic basis that is at least partially shared among affected individuals. If the cases share a set of genetic risk factors then, presumably, this means that the cases will be somewhat more closely related to each other, on average, than they are to control individuals. Devlin and Roeder then presented some numerical examples that suggested that cryptic relatedness may be an important effect in practice. However, it is difficult to know how realistic those examples are because they were constructed artificially, and were not based on a population genetic model.

At this time, there are few empirical data that bear on whether cryptic relatedness is a serious problem in practice. One study of association mapping in a founder population concluded that in that population, cryptic relatedness *did* have a significant impact on tests of association [10]. Methods exist that can incorporate kinship relationships into the test for association if such information is known [11–14]. If relationships are not known in advance, then genomic control methods can correct for cryptic relatedness [4,6,8], while structured association methods (developed for the population structure problem) cannot [7,9].

In this article, we aim to address the question of whether, and when, cryptic relatedness is likely to be a serious issue for case-control association studies. Our approach is to develop a formal model of cryptic relatedness within a population framework. We show that a natural measure of the impact of cryptic relatedness, that we will denote δ, depends on the population size, the genetic model parameterized by the recurrence risk ratio [15], and the number of sampled cases and controls. Our initial model assumes that studies are “well designed” in the sense that they do not have serious sampling biases, such as a bias toward enrolling related cases into a study. For that model, our results indicate that for association studies in large outbred populations, the confounding effect due to cryptic relatedness is expected to be negligible, but that it may well be a more serious issue in small, growing populations. We also consider two simple scenarios in which the sampling is biased toward collecting relatives among the cases. Such sampling can lead to non-trivial inflation.

## Results

### A Model of Cryptic Relatedness

Consider a study in which *m* cases affected with a disease and *m* random controls are genotyped at a single bi-allelic locus with alleles *B* and *b* that are at frequencies *p* and 1 − *p,* respectively. We aim to model the impact of cryptic relatedness on a test of association at this locus, assuming that the locus is not in fact linked to any disease-associated genes. The starting point for our notation and modeling is taken, with some modification, from [4].

We suppose that cases and controls are sampled from a single population (i.e., without population structure) of finite size, with discrete generations, and that mating is independent of the phenotype of interest. All individuals are sampled from the current generation. Since the impact of cryptic relatedness is due to alleles that are identical by descent, it will be necessary to model the coalescence times of chromosomes. We will use *T* ∈ {1, 2, 3, …} to denote the random time at which a particular pair of chromosomes in the current generation coalesces. (That is, *T* is the number of generations before the present at which the copies of the marker locus on each of the two chromosomes in question trace their ancestry back to a single ancestral chromosome.) According to standard models, for randomly chosen chromosomes (i.e., unconditional on phenotype) 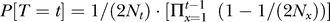 , where *N_x_* is the number of diploid individuals in generation *x* [16].

We will also assume that affected individuals have the same distribution of family sizes as do unaffected individuals, and that selection against the disease phenotype is negligible. Hence, chromosomes from affected individuals coalesce with chromosomes from random individuals at the same rate as do chromosomes from pairs of random individuals. To be precise, let *T*_(*i*,*a*)(*i′*,*a′*)_ denote the coalescence time between chromosomes *a* and *a′* from individuals *i* and *i′*. (Here, *a* and *a*′ denote one of the two copies of each chromosome, chosen at random in individuals *i* and *i′,* respectively.) Then by assumption,


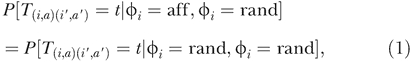


where Φ_*i*_ = aff and Φ_*i*_ = rand indicate that individuals *i* and *i′* carry affected and random (unknown) phenotypes, respectively. In contrast, we will show that chromosomes from pairs of affected individuals have an excess probability of very recent coalescence. The extra relatedness of cases occurs because they share a heritable trait, and not from average differences in the family sizes of affected and unaffected individuals. Under the assumption in Equation 1, it follows that *P*[Φ_*i*_ = aff|*T*_(*i*,*a*)(*i′*,*a′*)_ = *t*,Φ_*i*_ = rand] = *K_p_*, where *K_p_* denotes the overall population prevalence of the disease of interest. This is reasonable, because simply knowing that individual *i* has a relative *i′* whose affection status is unknown, should not alter the probability that *i* is affected.

We also define a quantity *K_t_* that is analogous to the standard relative recurrence risk *K_r_* [15]. Specifically, for a pair of individuals *i* and *i′,* where *i* is affected, *K_t_* is defined as the probability that *i′* is also affected, given that a specific pair of alleles from the two individuals coalesces to a common ancestral chromosome *t* generations before the present (where the alleles are at a locus unlinked to any disease loci): *K_t_* = *P*[Φ_*i′*_ = aff|Φ_*i*_ = aff, *T*_(*i*,*a*)(*i′*,*a′*)_ = *t*].

Notice, however, that the definition of *K_t_* implies some ambiguity in the actual relationship between the two individuals in question: e.g., *T* can be 1 either for siblings or for half-siblings, and 2 for cousins or half-cousins. Therefore, to evaluate *K_t_,* it will be necessary to be specific about mating patterns in the population. Later in the paper, we describe results for two particular models of random mating.

The ratio *K_t_/K_p_* will be denoted *λ_t_*. This is closely related to the standard recurrence risk ratio *λ_r_* [15], and measures the proportional increase in risk for an individual given that one of his/her chromosomes coalesces with the chromosomes of an affected individual *t* generations before the present. Due to shared genetic or environmental factors, *λ_r_* (and hence *λ_t_*) is often >>> 1 for close relatives; this means that even random sampling of affected individuals can lead to a sample that contains an excess of related cases.

Let 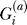 be an indicator variable for the presence (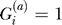 ) or absence (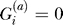 ) of the *B* allele on the *a*th copy of this locus in affected individual *i.* (Here, *a* ∈ {1, 2} labels the two homologous copies of a marker in a diploid individual.) Similarly, 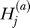 denotes the analogous indicator variable for the *a*th copy in control individual *j.*

Then we define a test statistic, *D,* which measures the difference in the overall allele counts between case and control samples at a given marker:


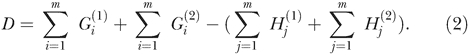


When appropriately normalized, *D* forms the basis of familiar tests of association. Under the null hypothesis, *D* ^2^/*Var*[*D*] is *χ* ^2^ distributed with one degree of freedom [4]. *D* is proportional to both the trend test [17] and to the allele test [18].

Under the standard null hypothesis, an allele copy at a given marker is type *B* with probability *p, independently for all allele copies in the sample*. The independence assumption implies that there is no population structure, no inbreeding, and that all cases and controls are mutually unrelated. If all alleles are mutually independent, then the variance of *D* is 4*mp*(1 − *p*). If, however, cryptic relatedness exists in the sample, then the actual variance of the test—call this *Var* ^*^[*D*]—will exceed the variance predicted under the null hypothesis. We will measure the deviation from the null variance using the “inflation factor” *δ,* defined as follows:


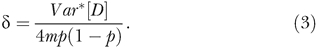


In the absence of true association between the marker and the genotype, the commonly used test of association, *D* ^2^/[4*mp*(1 − *p*)], has a distribution that is the product of *δ* and a *χ* ^2^ random variable [4].

Values of the inflation factor, *δ,* near 1.0 imply that the standard test of association is correctly calibrated, or nearly so. Values of *δ* substantially larger than 1.0 indicate that there will be an excess of false positive signals. Our target here is to derive an expected value for *δ* under a model of cryptic relatedness. These general results do not rely on a particular genetic model, but we do present examples using an additive model. We consider models of constant population size and of recent population expansion.

### Theory

We now characterize the extra variance that is caused by relatedness within a given case-control study, and use this to compute the expected inflation factor *δ*. Starting from the definition of *D,* in Equation 2, we can write *Var* ^*^[*D*] as


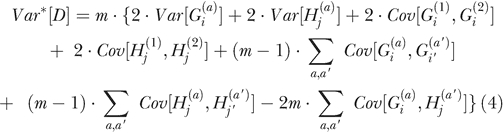


where *i* ≠ *i′, j* ≠ *j′.* We now need to determine how the value of this expression depends on cryptic relatedness.

Since *G_i_* and *H_j_* are Bernoulli trials, we have:


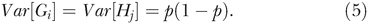


The following two terms in Equation 4 account for the possibility of departures from Hardy-Weinberg equilibrium in the sample. Assuming that these factors are independent of case-control status, we can write these as


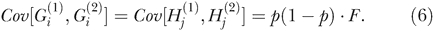


where *F* measures the extent of the departure from Hardy-Weinberg equilibrium [4,19]. If, in fact, there is a different average level of inbreeding in cases than in controls [20], then we would replace *F* in Equation 7 and thereafter, with an average *F* across the cases and controls. (Notice that, unlike here, the inflation factor used by Devlin and Roeder was defined relative to the trend test, so that Hardy-Weinberg departures cancel out in their formulation.)

In our model, the controls are sampled randomly from the population. This means that the terms 
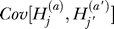
 and 
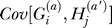
 are zero. This follows because, conditional on *p,* the fact that a random allele in the population is *B,* or *b,* provides no additional information about the genotype of another case or control in the sample. The assumption that controls are sampled randomly will usually be a good approximation, even if controls are specifically ascertained as not having the disease. As we will show below, the size of these covariance terms depends on the recurrence risk ratio for the phenotype, and the recurrence risk ratio for being unaffected is typically near one.

Next, since case alleles *G_i_* are each similarly distributed, we can reduce Equation 4 by characterizing a single covariance between case alleles and then collecting the sum of all covariance terms that contain only case alleles. Given this, the Hardy-Weinberg equilibrium terms, and Equation 5, Equation 4 simplifies to:


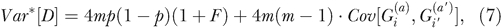


where *i* ≠ *i′*. And now, finally, we need to evaluate 
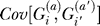
 under a model of cryptic relatedness. In order to do this, we first need to evaluate the probability that alleles in affected individuals share a common ancestor in generation *t* before the present. This will allow us to calculate the extra relatedness in cases due to the phenotype.

Recall that *K_p_* is the population prevalence of the disease; *K_t_* is the probability that a relative of an affected individual is also affected, given that the two individuals share a common ancestor *t* generations before the present; and that *λ_t_* = *K_t_/K_p_* is the corresponding ratio of risks [15]. Next, let *T*_(*i*,*a*)(*i′*,*a′*)_ denote the coalescent time of allele copies *a* and *a′* from individuals *i* and *i′.* In a slight abuse of notation, we will abbreviate *T*_(*i*,*a*)(*i′*,*a′*)_ as *T_ii′_*. In what follows, individuals *i* and *i′* are random (unphenotyped) draws from the population, except when specifically noted (e.g., Φ_*i*_ = aff indicates that *i* is affected). Then, using Bayes' rule, we can compute the coalescence rates for two chromosomes sampled from affected cases in the population as follows:


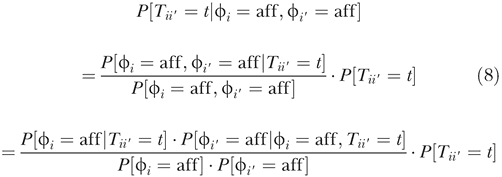


where *P*[*T_ii′_* = *t*] denotes the prior probability of coalescence in generation *t,* for random (unphenotyped) individuals. Next, using the assumption that affected and unaffected individuals coalesce with random chromosomes at the same rate (Equation 1), it follows that *P*[Φ_*i*_ = aff|*T*_ii′_ = *t*] = *K_p_*, and hence


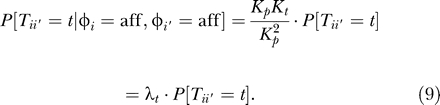


Equation 9 produces a pleasingly simple result: the coalescence rate for chromosomes from affected individuals is increased by a factor that is closely related to the standard recurrence risk ratio.

### Recurrence Risk for Relatives

The recurrence risk ratio is an important quantity in genetic epidemiology, and is widely measured [1]. For siblings, typical recurrence risk ratios for complex diseases range from around 2 to 50. For more distant relationships, the risk ratio declines approximately geometrically toward 1 as the number of meioses separating two relatives increases.

In our theoretical development, we will assume that disease inheritance is governed by a single additive gene [15], unlinked to the marker locus of interest. Other genetic models, including more complex models, behave similarly to this, except that the rate of decay of *λ_t_* with increasing *t* may differ somewhat [15], leading to different coefficients in the cryptic relatedness term in Equation 16 below.

For the additive model, [15] obtained an expression for the recurrence risk ratio, *λ_r_,* for any possible relationship, *r,* in terms of the recurrence risk ratio for full siblings, *λ_s_:*


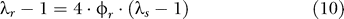


where Φ_*r*_ is the kinship coefficient between *r*th-degree relatives. For example, Φ_*r*_ = 1/4 between sibs, and decays by 1/2 for each increment to *r*. To connect *λ_r_* to our model—which is written in terms of coalescent time *t* instead of *r*—we need to be more explicit about the mating patterns in the population model.

For example, under the standard Wright-Fisher model where individuals select their parents independently at random, most relatives are “half-relations”: half-siblings, half-first cousins, half-second cousins, etc. In that case, for *t* = 1, 2, 3, … , the corresponding kinship coefficients are Φ_*r*_ = 1/8, 1/32, 1/128, and so on. Then for example, for *t* = 2, *λ_t_* − 1 = 4(*λ_s_* − 1)/32. If instead, mating is purely monogamous, but partners are still chosen at random, then all relationships are “full”: full siblings, full cousins, etc. That is, for *t* = 1, 2, 3, … , the corresponding kinship coefficients are Φ_*r*_ = 1/4, 1/16, 1/64,… .

In summary, *λ_t_* may be much larger than 1 for the closest relatives, but it becomes approximately 1 if the common ancestor is more than just a few generations ago (> 10 or 15, say). This qualitative conclusion does not depend strongly on the assumed genetic model. Referring to Equation 9, this means that chromosomes from affected individuals have an excess probability of coalescing extremely rapidly (within the past few generations). If they do not, then they behave essentially like random chromosomes, for which coalescence takes place on timescales of thousands of generations in typical populations (Figure 1).

**Figure 1:**
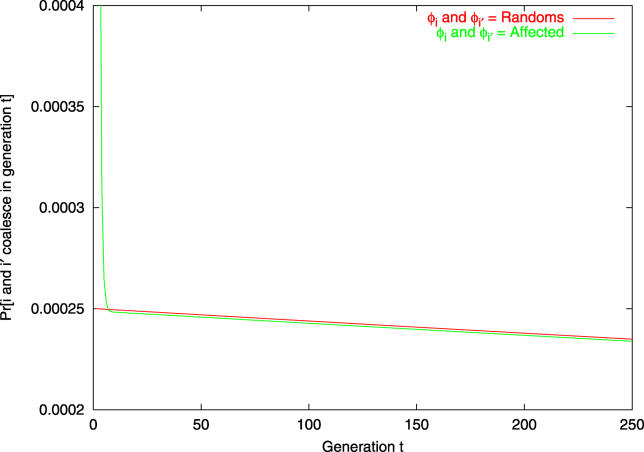
Coalescence Rates for Pairs of Random Chromosomes (Red) and for Pairs of Chromosomes from Affected Individuals (Green) Notice that chromosomes from affected individuals have a small excess probability of coalescing very rapidly (i.e., in the most recent ten generations or so). Otherwise, their coalescence rates are essentially like those of random chromosomes. The region at the left-hand side of the graph between the red and green lines represents the excess probability of very recent coalescence among case chromosomes (denoted *R* in the text). This is what gives rise to the effect of cryptic relatedness. For larger *t,* the line for cases drops slightly below the line for random individuals, since both distributions integrate to 1. These plots assume an additive genetic model, with *λ_s_* = 60, the “half”-relationships mating model, and a population size of 2,000. The line for cases was generated under the approximation that the excess relatedness is completely limited to the first *n* = 10 generations. In this case, the maximum coalescent probability for case chromosomes is 0.00275, when *t* = 1; R ≈ 0.00334. As expected, the mean coalescence time is ≈ 4,000 generations for both distributions. Alterations in *n* yield similar results (unpublished data).

The dynamics of this process are reminiscent of structured coalescent models with many demes [21–23]. In those models, two chromosomes from the same deme either coalesce with each other very quickly or escape into the population at large, and coalesce on a much longer time scale. These two phases have been described by John Wakeley as the “scattering phase” and the “collecting phase,” respectively [24]. An extreme example of this type of process (with selfing) was illustrated by Rousset [25].

### Calculating the Inflation Factor

As described above, ancestral chromosomes of affected individuals coalesce at an increased rate during the most recent few generations (Figure 1), and otherwise behave essentially like random chromosomes. We now provide a heuristic derivation of the inflation factor *δ;* later we show that our expression closely approximates the results obtained in simulations. For simplicity, we consider the following approximation.

Let *R* be the *excess* probability of very recent coalescence for affected chromosomes relative to random chromosomes. That is,


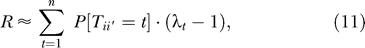


where *n* might be taken as 10 or 15, say. Then write:


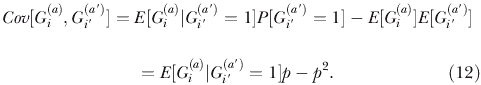


To evaluate 
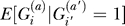
 , notice that there are two cases. With probability *R,* the two chromosomes coalesce very rapidly due to their shared phenotype. In that case, they share such a recent common ancestor that they are almost certainly identical by descent. In the second case, with probability 1 − *R,* the two chromosomes behave as random chromosomes, and their genotypes are independent Bernoulli draws from the population frequencies:


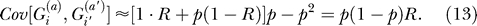


And finally, substituting Equations 11, 13, and 7 into Equation 3, we obtain


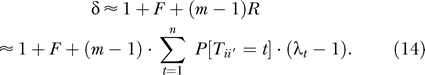


Equation 14 is worthy of discussion. When the simplest model of independence among sampled alleles holds, then *δ* = 1. The term containing *F* corresponds to Hardy-Weinberg departures, due to inbreeding for instance. The summation term corresponds to the effect of cryptic relatedness; the sum itself can be thought of as calculating the excess probability of identity by descent between chromosomes from affected individuals. Overall, the effect of cryptic relatedness increases linearly with the sample size *m* (for a given population size and *λ_t_*).

### Applications to Specific Models

In this section, we evaluate Equation 14 under a range of specific models, in order to determine when cryptic relatedness is likely to have a substantial impact on case-control studies. The models presented assume an additive genetic model, as described above. At first, we will assume that the population is of constant size *N,* so that the probability of coalescence in generation *t, P*[*T_ii′_* = *t*], is (1 − 1/2*N*)^t−1^/(2*N*). After that, we turn to models with population growth. For simplicity, we set *F* = 0.

### The Inflation Factor in Populations of Constant Size

Recall from Equation 10 that λ_*t*_ − 1 = 4Φ_*r*_(λ_*s*_ − 1). Recall also, that when individuals select their parents independently at random, as in the standard Wright-Fisher model, that most relatives are “half-relations” (e.g., half-siblings, half-cousins, etc.), and then the kinship coefficients Φ_*r*_ are 1/8, 1/32, 1/128, … for *t* = 1, 2, 3, etc. Using *δ*_half_ to indicate this situation where individuals are related via “half-relationships,” it follows that


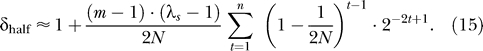


Noting that 
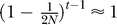
 for small *t* (provided that *N* is not small), and that ∑ 2^−2*t*+1^ converges quickly to 2/3; Equation 15 can be further approximated as


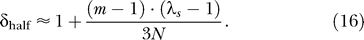


If instead, mating is purely monogamous, but partners are still chosen at random, then all relationships are “full”—e.g., full siblings, full cousins, etc., and the kinship coefficients are two-fold higher. The corresponding inflation factor, *δ*_full_, is


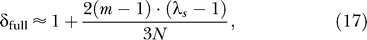


indicating that the impact of cryptic relatedness is approximately doubled when there is fully monogamous pairing of parents, compared to when there is independent pairing of parents for each offspring.

### Simulations

To check the accuracy of our analytical results, we generated population histories via Wright-Fisher simulation and estimated the inflation factor, *δ,* for a given disease and population genetic model, as described in the Materials and Methods section. Results are presented in Table 1, and compared to predicted results from Equation 16. The results show close agreement between the analytical prediction and the simulation results. In some cases, the analytical results slightly overestimate the inflation factor, probably due to the approximations used in relating Equation 9 to *δ*.

**Table 1:**
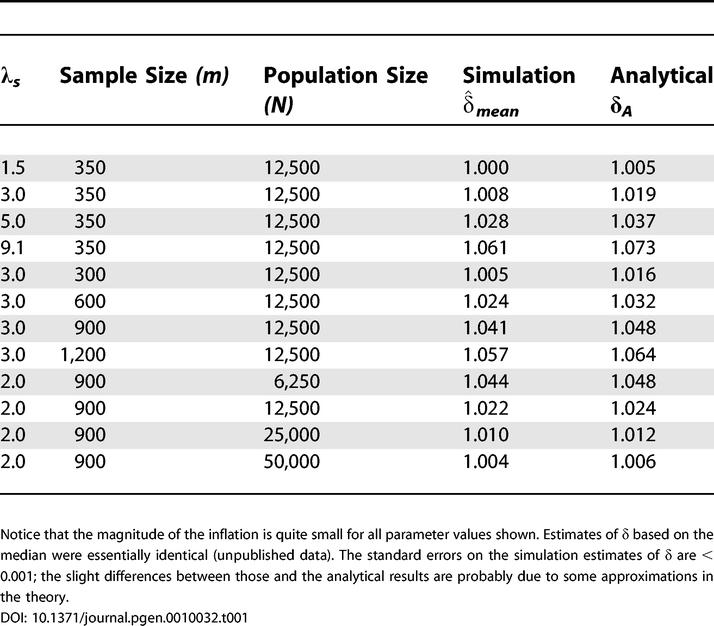
Values of the Inflation Factor as a Function of Model Parameters, and a Comparison of the Simulated (δ̂_*mean*_) and Analytical (*δ_A_*) Predictions, for Populations of Constant Size

While the choice of an additive model for the phenotype (i.e., a heterozygote has exactly one-half the penetrance for the phenotype as a homozygote for the risk allele does) is mathematically convenient, alternative modes of inheritance (including multilocus models, or models with dominance components) are certainly likely in practice. Such models will have the impact of changing the rate of decay of *λ_t_,* and hence the coefficient of the cryptic relatedness term in Equations 16 or 17. While we do not present a complete exploration of such models, we have performed a modest number of additional simulations under non-additive models. We have found that those results are qualitatively similar to the results presented above (unpublished data).

### Intrinsic Constraints on *δ*

Table 1 shows the predicted impact of cryptic relatedness for a range of possible disease parameters. The magnitude of the inflation factor is fairly small for all parameter combinations shown, with a maximum value of 1.07. To make this more concrete, an inflation factor of 1.07 implies a quite modest excess of false positives: for instance, a fraction 1.5 × 10^−3^ of tests would be significant at the *p* = 10^−3^ level. As another example, consider a genetic model based loosely on a study of autism [26], where *λ_s_* = 75, and *K_p_* of 0.0004. Assuming the full-sibling model of relatedness, a sample size of 1,000, and a population size of 2.5 million (i.e., the number required to find that many cases), *δ* is just 1.02.

These examples notwithstanding, however, Equations 16 and 17 seem to suggest that *δ* can be made arbitrarily large simply by increasing the sample size *m*. But in fact, the space of sensible models is actually rather constrained. Since *m* cannot exceed *K_p_* times the population size, there is a practical limit on *m* for a given *λ_s_* and population size. Because of this constraint, it is difficult to construct biologically plausible parameter combinations that result in substantial inflation factors for randomly mating populations of constant size.

To be more specific, let *K_s_* be the rate of disease in full siblings of an affected proband, i.e., *K_s_* = *λ_s_ K_p_*. Furthermore, let *f* be the fraction of all affected individuals in the population that are included in the sample. Then, noting that *f* = *m/NK_p_,* Equation 17 can be rewritten as


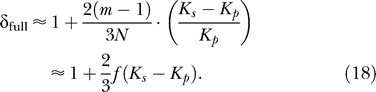


Therefore, since *f* ≤ 1, for diseases where *K_s_* is smaller than, say, about 1%, the inflation factor is negligible. The only way to get large values of *δ* is to have high values of *K_s_* − *K_p_* and nearly complete ascertainment of cases (high *f*). For instance, if *K_s_* were 0.2 and *λ_s_* were 4, then the inflation factor could be as large as 1.1, producing a small excess of false positives. But the latter calculation assumes complete sampling of affected individuals (*f* = 1), which would usually be difficult for a common disease.

In summary, in populations of constant size, the impact of cryptic relatedness is generally very small, unless (1) *K_s_* is quite large—more than 0.2, say, and (2) *f* is near 1, meaning that there is nearly complete ascertainment of cases from the population. Hence, cryptic relatedness should not be a serious concern for most complex trait studies in stable populations, assuming random sampling of cases. As we will show in the next section, the situation is more serious for models with population growth.

### The Inflation Factor with Changes in Population Size

We now consider a model that allows for changes in population size. Let *N_t_* represent the population size at time *t*. Then, provided that the coalescent probability 1/2*N_t_* is not especially large in any of the recent generations, and since *λ_t_* − 1 decays as *t* increases, we can rewrite and simplify Equation 14 to


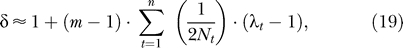


where again *λ_t_* refers to the recurrence risk ratio for coalescence time *t*. Because (*λ_t_* − 1) decays quickly toward zero, it is apparent that only changes in population size during the last few generations will impact *δ*. Moreover, for given values of *m* and *λ_t_,* smaller population sizes in the past will produce higher inflation factors.

To check the accuracy of our results regarding demographic expansion, we modified the forward simulation procedure used above such that instead of a single *N,* we simulated exponential growth that began at time *t*_onset_ in the recent past starting at an initial population size *N_A_*. For each subsequent generation *t,* the population size was determined by the equation *N_t_*_+1_ = *N_t_ · e^α^* for a growth rate *α* such that the population size in the final generation is *N_f_*. We performed at least 10,000 repetitions for each parameter combination, and the 95% standard error about the mean for each estimated *δ* was no greater than 0.01. In our analytic calculation, we assumed the “half” relationships model, as in Equation 15 and 16.

Results of the simulations, for a range of parameter values, are summarized in Table 2. Again, the theoretical prediction in Equation 19 is close to the simulated values. Under very recent growth models, δ̂ can be substantial (as much as 2.5 for the extreme growth scenario shown). Under more realistic models of population growth, the effect of cryptic relatedness is smaller, but still non-trivial. Based on these results, it seems clear that the magnitude of growth is an important factor for determining *δ*. In populations that have grown rapidly from small size in the past few generations, cryptic relatedness may indeed lead to high inflation factors. It should be noted that many of the models presented have extreme growth; hence, the higher levels of cryptic relatedness shown here are likely to exceed anything seen in practice in human populations.

**Table 2:**
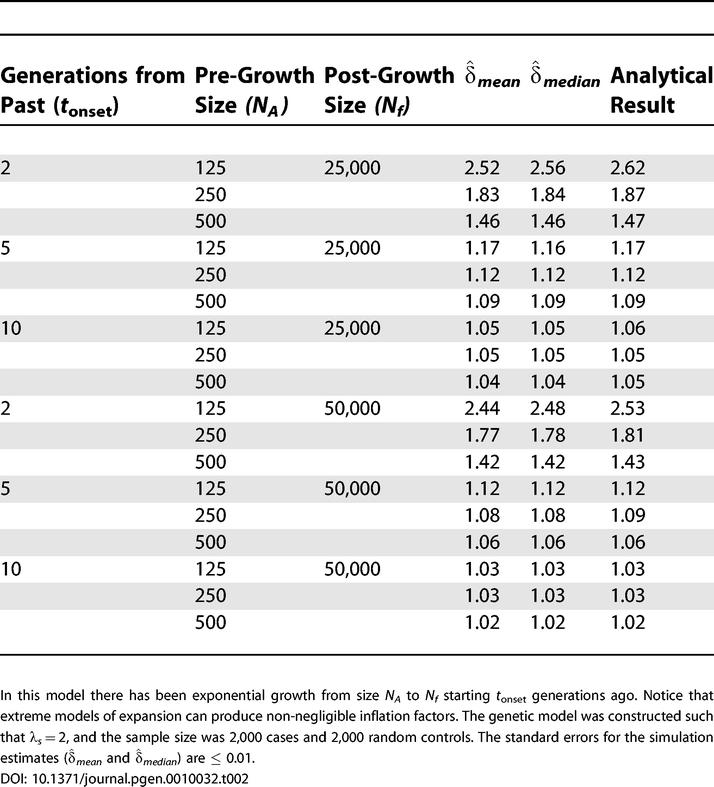
Values of the Inflation Factor in Very Recently Expanded Populations

**Figure 2:**
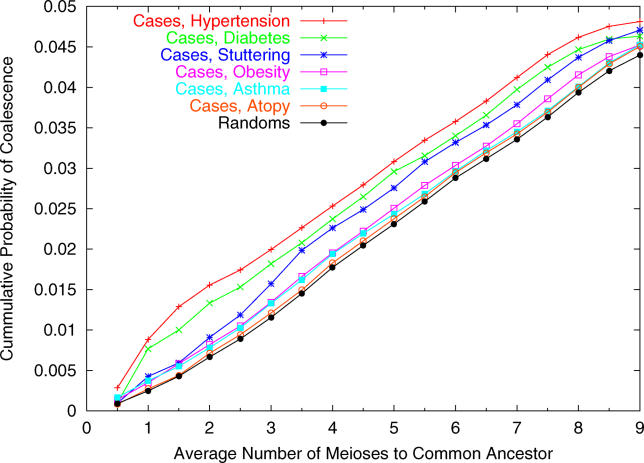
Cumulative Probability of Coalescence within the Last *n* Meioses in the Hutterite Founder Population Each line plots the estimated probability that two chromosomes drawn at random, from different individuals affected with a given phenotype, or from two random control individuals, descend from a single ancestral chromosome within the last *n* meioses. These estimates are based on the recorded Hutterite genealogy. The *x*-axis plots the average number of meioses along the two lineages back to the common ancestor. Notice that in the most recent generations, the case samples coalesce at higher rates than do random controls.

The qualitative difference between the equilibrium model and the population-growth model can be understood as follows. Consider two studies in which *m* affected individuals are sampled from each of two populations that have the same current size. If one population is of fixed size, while the other has grown rapidly from a smaller size, then the probability that two individuals are closely related is much higher in the growing population than in the equilibrium population. It follows from Equation 19 that this produces a higher inflation factor in the growing population than in the stable one.

### Cryptic Relatedness with Biased Sampling

Thus far, we have considered models that assume “good” sampling design, in the sense that the sample of cases represents a random sample of the affected individuals in a population. We now consider the impact of sampling schemes that bias toward enrolling close relatives as cases in a study. For the previous models, we showed that with random ascertainment of cases, the inflation factor *δ* is maximized with complete ascertainment of cases from a population. The following models are instead motivated by the scenario in which a study enrolls only a small fraction of the affected individuals in a large population but, due to sampling biases, tends to recruit close relatives. Such situations might arise in practice if, for example, a patient at a clinic or in a study encouraged affected family members to visit the same clinic, or also to enroll in the study.

As an extreme, but simple example, consider first the situation in which the case sample consists of *m*(1 − *σ*) unrelated affected individuals, plus *mσ*/2 pairs of affected siblings (σ ∈ [0, 1]). The controls are all unrelated to anyone else. Assume furthermore that there is not inbreeding, so that *F* = 0 and the probability of recent identity-by-descent for chromosomes in siblings is 0.5. (For simplicity, we assume both in this and the next model that the sampling is from a sufficiently large population relative to *m* that we can approximately ignore the impact of cryptic relatedness apart from that induced by the biased sampling of siblings.) Then recall from Equation 14 that *δ* ≈ 1 + *F* + (*m* − 1)*R* where *R* is the (average) excess probability of recent coalescence, computed across all pairs of case chromosomes. In this model, a fraction *σ*/(*m* − 1) of the pairs of individuals are siblings. The probability that a randomly selected chromosome *a* in one sibling and *a′* in the other sibling descend from the same parental chromosome is *R* = 1/4. Hence, for this model we obtain *δ* ≈ 1 + *σ*/4. At most, if the entire case sample is made up of sibling pairs, *δ* = 1.25. Any relatedness among the controls would further increase *δ*.

As a second simple example, suppose that a study recruits only a small fraction of affected individuals from a large population, but that recruits sometimes then encourage their siblings to enroll. Let the number of siblings of a recruited individual be Poisson with mean *g,* and let *h* be the probability that an affected sibling goes on to enroll in the study, independently for each affected sibling. Then the number of siblings of the initial recruit who enroll as patients in the study is Pois(*ghK_s_*). After some algebra, it follows that the expected fraction of pairs of case individuals in the sample who are siblings is *γ*(*γ* + 2)/[(*m* − 1)(*γ* + 1)], where *γ* = *ghK_s_*. Hence (again taking *F* = 0), we obtain


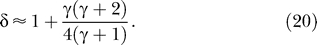


From these examples, it seems that biased sampling of cases can have a substantial effect on inflating the test statistics—though this is less dramatic perhaps than might have been expected. For example, suppose that index cases have an average of *g* = 2 siblings, that they refer affected siblings with probability *h* = 0.5, and that *K_s_* = 0.4. Then the inflation factor *δ* ≈ 1.17.

### Cryptic Relatedness in the Hutterites

We have used data collected from a founder population, the Schmiedeleut (S-leut) Hutterites of South Dakota, to illustrate the impact of cryptic relatedness on association studies for phenotypes measured in that population [27]. The S-leut Hutterite population consists of 13,000 members connected by a single, known, multigenerational pedigree that goes back to 64 founder individuals about 12–13 generations ago. Approximately 800 members of this population have been phenotyped for many traits and genotyped at a large number of microsatellite markers [27,28]. We considered six phenotypes: asthma, atopy, diabetes, hypertension, obesity (> 33% body fat for males, > 38% body fat for females), and stuttering (ever stuttered), all of which we treated as binary traits. We are grateful to C. Ober, who kindly allowed us access to these data.

It has previously been reported that naïve tests of association produce an excess of false positive signals in this population [10,14]. Our aim in this section is to further explore the impact of relatedness among cases in the context of the theory developed here. In particular, we set out to determine (1) whether we could detect excess relatedness among affected individuals, (2) the empirical level of confounding at random markers, and (3) whether we could predict the observed level of confounding based on the pedigree.

The fact that we have complete genealogical information for the Hutterites allows us to estimate the coalescence probabilities for pairs of alleles in any two individuals at any time since the founding of this population. These probabilities were estimated as described in the Materials and Methods section. The data do not provide information about coalescent events more than about 12 generations before the present, but the theory presented above suggests that the impact of cryptic relatedness is due to very recent coalescent events (and this is supported by our results, as follows).

The results of this analysis are presented in Figure 2. For all six phenotypes, there is an excess rate of coalescence within the pedigree, relative to random controls. Moreover, most of the increased probability of coalescence is due to rather close relatedness among cases (i.e., mainly for ≤ 4 meioses). This is consistent with the theoretical prediction that *λ_t_* − 1 declines rapidly to zero.

We next used the genotype data to obtain an empirical estimate of *δ* for each phenotype, under the assumption that most random markers are not genuinely associated with disease loci. We considered 437 microsatellite markers typed in approximately 800 members of this population and estimated *δ* as described in the simulation methods above. The procedure for estimating *δ* in this data is described in the Materials and Methods section.

Table 3 summarizes the results from this analysis. For all six phenotypes, there is a non-trivial inflation to the test for association under the null hypothesis, in the range of about 1.2–1.3. This is consistent with the previous report by Newman et al. of an excess of positive signals at a set of microsatellite markers in this population [10]. An inflation factor of 1.2 implies a rejection rate that is ≈ 1.5-fold too high at the 5% level, and ≈ 2.7-fold too high at the 0.001 level. A *δ* of 1.3 implies a rejection rate that is ≈ 1.7-fold too high at the 0.05 level, and ≈ 3.8-fold too high at the 0.001 level. In a majority of cases, the predicted level of inflation matches empirical estimates, and the analytical result in all cases predicts a non-trivial inflation factor for each phenotype. For related subsets of phenotypes (asthma/atopy and obesity/hypertension/diabetes), the observed inflation factor appears similar. However, this is partly coincidental: *δ* depends on both the coalescent time and the sample size, which are different for each phenotype.

**Table 3:**
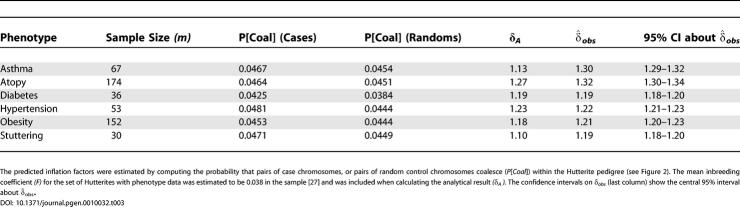
Observed (δ̂*_obs_*) and Predicted (*δ_A_*) Inflation Factors for Six Phenotypes Measured in the Hutterite Founder Population

## Discussion

Should one be concerned about confounding from cryptic relatedness in association studies? To address this question, we have developed theory to predict the amount of cryptic relatedness expected in a random-mating population. Our results demonstrate that confounding effects of this kind are expected to be substantial only under rather special conditions. The bulk of the effect is due to the occurrence of quite close relationships among sampled individuals. Except in small populations, random pairs of affected individuals are unlikely to be closely related. Our results in Equation 14 show that for a given genetic model and population size, the impact of cryptic relatedness grows linearly with sample size. However, this obscures the fact that in practice, the maximum number of cases *m* that can be sampled from a given population size*, N*, is constrained by the population prevalence (*K_p_*), and hence is inversely related to *λ_r_*. That is to say, assuming constant population size, it is difficult to construct examples in which cryptic relatedness has an appreciable effect.

In contrast, studies of populations in which there has been rapid and recent population growth, and where the total study population is small, should indeed be concerned about cryptic relatedness. This scenario produces higher levels of relatedness than are possible for the same values of *m* and *λ_r_* in stable populations. Studies in populations that meet these conditions—especially founder populations—should use pedigree-based methods or genomic control to minimize false positives due to cryptic relatedness [4,10,12].

Another situation in which cryptic relatedness may be important is when there is extensive inbreeding. A model in which individuals are likely to mate with relatives will increase *δ* relative to the models analyzed in this paper. When there is inbreeding, if two individuals share one recent common ancestor, they are likely to share other recent ancestors. That is, conditional on having a recent common ancestor, the expected kinship coefficient between two individuals would be higher than modeled in Equations 16 and 17. With modest inbreeding, this is likely to be a small effect, but the effect may be important in some populations with extensive inbreeding. Indeed, population structure may be viewed as a strong form of inbreeding, and that is often suspected to be a non-trivial source of confounding [29]. In contrast, sampling schemes that draw both cases and controls equally from just a segment of a population (e.g., from part of a city) should not induce particular problems. Even if there is extra covariance among sampled individuals, this should occur both within and between cases and controls equally, and thus cancel (Equation 4).

It should be noted that our results assume that the disease phenotype is selectively neutral (see discussion surrounding Equation 1). If, in fact, affected individuals or mutation carriers have fewer offspring than normal, then this will mean that affected individuals tend to have fewer close relatives than do random individuals. This effect would in many cases lower the probability of recent coalescence of case chromosomes, thus reducing the size of *δ*. This situation would reduce the level of cryptic relatedness relative to the models presented here. Conversely, a phenotype that increased fitness (perhaps in carriers of genes responding to selection only) might lead to increased *δ*.

Lastly, it should be noted that our primary model assumed a “good” epidemiological design in which individuals are ascertained randomly from the population. However, cryptic relatedness can also result from the non-random ascertainment of family members in a case-control study. For instance, affected family members might be more likely to seek treatment in the same clinic, or affected individuals might encourage their affected relatives to enroll in a study. These types of situations may be difficult to detect at the time of enrollment, but can have non-trivial consequences even in large outbred populations. We have shown that these situations indeed result in excess false positive rates. After data collection, we recommend the use of techniques for identifying cryptic relative pairs based on genetic data [30–33]. Genomic control [4] can then be helpful for identifying any residual inflation.

## Materials and Methods

### Simulations.

To check the accuracy of our initial analytical results, we generated population histories via Wright-Fisher simulation and estimated the inflation factor, *δ*. A population of size *N* was advanced forward in time 4*N* generations, with non-overlapping generations and random pairing of parents, independently for each offspring. For each simulation, 1,000 bi-allelic sites separated by a recombination fraction of 0.5 (i.e., freely recombining) were simulated with a mutation rate of *θ* = 4*Nμ* = 1. After 4*N* generations, a random site with the desired allele frequency was selected as the true disease locus, and affection status was assigned to all members of the population based on an additive genetic model. To shorten the computational time, we initiated the simulations such that a smaller population with proportionally higher mutation rate was advanced forward in time until a given point in the distant past, and then the population size and mutation rate were rescaled to the desired levels. Samples of *m* random controls and *m* affected cases were then drawn from the simulated population. Then, for each marker, apart from the disease locus, we constructed the 2 × 2 contingency table containing the allele counts for cases and controls, respectively; provided that the expected count for each cell in the table was at least five, we computed the standard Pearson's *χ* ^2^ test statistic. We then estimated the inflation factor *δ* using estimators based on both the mean and median values of the *χ* ^2^ statistics [4,6]. For each estimated *δ,* 95% standard errors about the mean were based on 10,000 replicate simulations.

### Estimating coalescent probabilities in the Hutterites.

We estimated the coalescent probabilities for pairs of alleles in two individual Hutterites by the following. Starting from the affected individuals in the population, or from a matched random sample of individuals from the current population, we simulated the inheritance of a pair of randomly chosen chromosomes from different individuals, backward through time, from the present to the founders of the population. If the two chromosomes coalesced to a common ancestral chromosome within the pedigree, we counted the number of meioses back to that common ancestor, reporting the average number if the number of meioses was different on the two lineages. We repeated this procedure until we observed at least 500,000 coalescence events within the simulation. To estimate the mean inbreeding coefficient (*F*) in this sample, we used the same procedure as above except that we picked the two chromosomes from the same random individual, traced them backward in time, and determined how frequently those two chromosomes coalesced within the pedigree.

### Calculating the inflation factor in the Hutterites.

For each marker, we constructed a 2 × *k* contingency table, where *k* was the number of alleles for this marker. Then, we pooled the smallest allele counts in the table with the second smallest allele counts until a 2 × 2 contingency table was formed. These artificial 2 × 2 tables should mimic the results that would be obtained using bi-allelic markers. The depth of the pedigree is short enough that mutation within the pedigree should have minimal impact on *δ*. For each phenotype, we selected a random sample of controls with data collected for the analyzed phenotype and then treated the remaining affected individuals in the sample as cases. The list of random controls was then truncated (randomly) so that the sample sizes were equal in the two groups. For this set of cases and controls, we estimated *δ* based on the mean of tests from these 437 markers. This procedure was performed 1,000 times.

To be more careful about the possibility that some loci might be genuinely associated with a phenotype or in various degrees of linkage, we repeated the analysis using approximately 40 microsatellite markers, unlinked either to one another or to candidate gene regions showing evidence of linkage. The resulting δ̂s based on the mean were almost identical for all phenotypes to the larger marker sample (unpublished data). Finally, we generated a semi-analytical result for the phenotype by plugging the coalescent probabilities estimated from the pedigree, along with estimated inbreeding coefficients, and the average number of cases selected across all replicates, into Equation 14.
